# Subjective perception of spinal deformity after selective versus non-selective fusion of Lenke 1C curves

**DOI:** 10.1007/s43390-022-00479-8

**Published:** 2022-02-08

**Authors:** Davide Bizzoca, Andrea Piazzolla, Giuseppe Solarino, Lorenzo Moretti, Biagio Moretti

**Affiliations:** 1 UOSD Spinal Deformity Center, AOU Consorziale “Policlinico”, Bari, Italy; 2 Orthopaedic and Trauma Unit, Department of Basic Medical Sciences, Neuroscience and Sense Organs, Spine Unit, University of Bari Aldo Moro, AOU Consorziale “Policlinico”, Piazza Giulio Cesare 11, 70214 Bari, Italy

**Keywords:** Adolescent idiopathic scoliosis, Posterior spinal instrumentation and fusion, Selective fusion, Quality-of-life profile for spinal deformities (QLPSD), Spinal appearance questionnaire (SAQ), Revised scoliosis research society-22 questionnaire (SRS-22R)

## Abstract

**Purpose:**

To assess the self-image perception and the Quality-of-Life (QoL) in female adolescents, with Lenke 1C scoliosis curves, treated with selective versus non-selective posterior spinal instrumentation and fusion (PSF).

**Methods:**

Patients undergoing PSF for idiopathic adolescent scoliosis (AIS) were recruited and divided into two groups: patients managed with selective thoracic fusion (STF) were included in Group A, whereas patients treated with non-selective fusion (N-STF) in Group B. Each patient completed the Italian version of the Scoliosis Research Society-22R questionnaire (SRS-22R), the Quality-of-Life Profile for Spinal Deformities questionnaire (QLPSD) and the Spinal Appearance Questionnaire (SAQ), before surgery and at 24-month follow-up.

**Results:**

One hundred and fifty seven female patients (mean age 16.38) were included in this study. 80 patients underwent STF, while 77 patients received N-STF. At 24-month follow-up, patients managed with N-STF showed better SRS-22R self-image mean score (*p* = .012), SRS-22R satisfaction mean score (*p* = .033), QLPSD body image mean score (*p* = .005), but worse SRS-22 function mean score (*p* = .006) and QLPSD back flexibility mean score (*p* = .007), compared with patients who underwent STF. In terms of self-image perception, patients undergoing STF showed significantly worse SAQ total mean score (*p* = .002), SAQ appearance mean score (*p* = .001) and SAQ expectation (*p* = .001). We found a significant correlation between SAQ appearance mean score and SRS-22R self-image (*R* = − 0.721), SRS-22 mental health (*R* = − 0.8), QLPSD psychosocial functioning (*R* = 0.7) and QLPSD back flexibility (*R* = 0.8).

**Conclusion:**

Although the STF of Lenke 1C curves provides better functional outcomes, in the present study, female patients receiving STF revealed a worse perceived body image, compared with patients treated with N-STF, at 24-month follow-up. Particular attention should be addressed to the preoperative patient’s mental health and body image perception, when choosing between STF and N-STF.

## Introduction

The surgical treatment of adolescent idiopathic scoliosis (AIS) aims at three-dimensional deformity correction, the achievement of a solid arthrodesis and prevention of the curve progression in future [1]. All these goals could be achieved with posterior spinal instrumentation and fusion surgery (PSF) with pedicle screws, which currently represents the gold standard for the surgical correction of AIS [2]. The main drawback of PSF surgery, however, is the sacrifice of motion in the fused lumbar segments, which consequently lead to a reduction of postoperative spinal mobility [3].

The most common scoliosis pattern in AIS is a right-sided major thoracic curve; frequently also a concomitant lumbar curve coexists. If this lumbar curve is nonstructural, it could be excluded from the fusion area, thus a selective thoracic fusion (STF) could be performed [4]. STF should be accurately planned to obtain a valid three-dimensional spinal alignment, avoid mechanical complications needing revision surgery and preserve spine flexibility.

The STF concept, introduced by Winter and Moe [5] in 1958 and further promoted by King et al. [6] and Lenke et al. [4], has been gaining increasing popularity in the surgical management of Lenke 1C curves, in the last decades. The STF success relies on the possibility to obtain a postoperative spontaneous lumbar curve correction, without including it in the fusion area, thus finally leading to a well-balanced spine.

Nonetheless, in daily clinical practice, several controversies exist in the choice of selective versus non-selective fusion, for the management of Lenke 1C curves [7], including both curve-related and patient-related factors suggesting a negative postoperative outcome [1, 7–11].

Although in recent years, several studies [9, 12–14] have attempted to identify preoperative radiologic factors able to predict the successful outcome of STFs, none of the previous studies, to the authors’ knowledge, has focused on the body’s image perception in adolescents undergoing posterior STF, compared with non-selective fusion (N-STF).

The present study aims to assess the body’s self-image perception and the QoL in adolescents with Lenke 1C AIS curves, treated with selective versus non-selective PSF, at 24-month follow-up. The secondary outcome is to identify any relationship between preoperative textual scales mean scores and postoperative self-image unsatisfaction, in adolescents undergoing STF.

## Materials and methods

### Study subjects and clinical evaluation

Patients who underwent posterior spinal instrumentation and fusion (PSF) for idiopathic adolescent scoliosis (AIS) between January 2013 and March 2018, at our Spinal Deformity Center were included in this retrospective observational study. Ethical clearance was obtained from our center’s Clinical Research Ethics Committee (Code: 6479), as per the 1964 Declaration of Helsinki. All the patients and their parents gave written informed consent before enrollment in the study.

Inclusion criteria: Lenke 1C AIS; main thoracic curve Cobb angle > 45°; gender: female; no prior spine surgery. Exclusion criteria: left convex thoracic scoliosis; a history of mental disorders; concomitant musculoskeletal diseases; neurological diseases; metabolic diseases; the presence of congenital spine deformities; spine revision surgery within the first 24-month after PSF.

The enrolled patients were divided, based on the selective or non-selective PSF, into two groups: in Group A were recruited patients who underwent selective thoracic fusions—i.e., lower instrumented vertebra (LIV) at L1 or more cephalic, while in Group B patients treated with non-selective fusion (N-STF), i.e., LIV at L3 or more caudal. The decision to perform a selective or non-selective spinal fusion was made by the senior spine surgeon, after a collegial decision-making process.

The following demographic and operative data were recorded: age, body mass index (BMI), Risser grade, pre-operative main thoracic curve and lumbar curve Cobb angles, pre-operative coronal balance, number of instrumented vertebrae, total operative time and hospital stay.

Each patient underwent postoperative clinical and radiological evaluations, according to the protocol used at our Institution. All radiographic measurements were obtained by two independent observers, using Surgimap (Nemaris Inc., Ver. 2.3.2, NY, USA), a validated software; the inter-observer concordance as assessed by the Cohen *K* statistic was high (0.9).

### Surgical procedure

All the PSF procedures were performed by the same senior spine surgeon (P.A.), assisted by a junior surgeon; the same anesthesiology team followed all the procedures. Cell salvage autologous blood recovery system was used during all surgical procedures [15].

All the patients underwent PSF surgery using a system of titanium roads and screws (Solera System, Medtronic, Minneapolis, USA). Bilateral pedicle screw insertion was performed at each instrumented level; the pedicle screws were implanted using the free-hand technique [16]. All the patients received a high-density pedicle screw construct. Facetectomies were performed on all instrumented vertebrae, to improve the curve correction. Spinal fusion was achieved through laminar decortication and autologous bone grafting, obtained from facet joints, spinous processes and decorticated laminae of each instrumented vertebra. An intradermic suture was performed in all the patients. No wound drains were placed in both groups.

### Patient-reported outcome measures

All the patients were assessed before surgery and at 24-month follow-up, using the following Patient-Reported Outcome Measures (PROMs): the Italian version of the revised Scoliosis Research Society-22 patient questionnaire (SRS-22R), the Quality-of-Life Profile for Spinal Deformities questionnaire (QLPSD) and the Spinal Appearance Questionnaire (SAQ). All the data were analyzed in a blind manner by an external statistician, who was unaware of the type of fusion (selective versus non-selective) received by the enrolled patients.

The Scoliosis Research Society-22R (SRS-22R) questionnaire consists of 22 items belonging to five domains, i.e., function, pain, self-image, mental health and satisfaction with management [17]. Each domain is composed of 5 items each, except for “satisfaction with treatment”, which has only two items. Each question is answered using a five-point Likert scale ranging from 1 (worst) to 5 (best) [17].

The Quality-of-Life Profile for Spine Deformities (QLPSD) contains 21 items grouped into five dimensions: psychosocial functioning, sleep disturbances, back pain, body image and back flexibility [18]. The QLPSD total score ranges from 21 (i.e., the best quality of life) to 105 (i.e., the poorest quality of life).

The Spinal Appearance Questionnaire (SAQ) aims to measure the patient’s perception of the spinal deformity [19]. It is composed of 11 items containing standardized drawings showing the varying severity of several components of spinal deformity (SAQ Appearance), followed by 22 questions referring to patients’ impressions regarding their appearance (SAQ-Expectation) [19]. Each question is answered using a five-point Likert scale, ranging from 1 to 5, with higher scores reflecting worsening deformity. The SAQ has a total possible score ranging from 14 (best score) to 70 (worst score).

### Statistical analysis

Statistical analysis was carried out using SPSS^®^ (version 23; IBM Corp, Armonk, NY). The Shapiro–Wilk test was conducted to verify the normal distribution of data. Paired *t* test was performed to assess within-group variability at 24-month follow-up, compared with baseline. Unpaired *t* test and Chi-square test were used to assess intergroup variability. Pearson correlation test was performed to assess any relationship between the number of instrumented vertebrae and PROMs and the relationship between SAQ Appearance mean score, SRS-22R domains mean scores and QLPSD domains mean scores in Group A patients.

The data are presented in terms of mean value and standard deviation (SD); a *p* value < 0.05 was considered significant.

## Results

The main data of the study are summarized in Table 1. A total of 157 patients (average age 16.4, range 13–19 years old), were included in the present study. 80 patients underwent selective thoracic fusion (Group A), while 77 patients received non-selective fusion (Group B). Preoperative main thoracic curve Cobb’s angle showed no significant differences between groups. Preoperative lumbar curve Cobb’s angle was significantly higher in patients who received non-selective fusion (Group B), compared with patients who underwent selective thoracic fusion (Group A; *p* = 0.001).

**Table 1:** Demographic, radiographic and operative data for selective (Group A) and non-selective fusion (Group B) cohorts

	Group A (selective fusion)	Group B (non-selective fusion)	*p* value
Patients (*n*)	80	77	–
Age			
Mean ± SD	16.5 ± 2.1	17.1 ± 2.8	.64^+^
Range	13–19	14–19	–
BMI (Kg/m^2^)			
Mean ± SD	21.6 ± 1.8	21.9 ± 1.5	.443^+^
Risser 4, *n* (%)	21 (70%)	17 (62.96%)	.031^§^
Risser 5, *n* (%)	9 (30%)	10 (37.34%)	.022 ^§^
Pre-op coronal Cobb angle (mean ± SD)			
Main Thoracic curve	63.3 ± 12.9	61.7 ± 14.2	.354^+^
Lumbar curve	32.6 ± 10.6	39.3 ± 15.3	.001^+^*
Pre-op MT curve flexibility (mean ± SD)			
Mean ± SD	27.4 ± 16.5	28.2 ± 17.4	.135
Pre-op lumber curve flexibility (mean ± SD)			
Mean ± SD	39.2 ± 18.7	40.5 ± 16.5	.363
Pre-op MT AVT (mean ± SD)			
Mean ± SD	44.4 ± 4.6	43.2 ± 8.3	.098^+^
Pre-op L AVT (mean ± SD)			
Mean ± SD	27.5 ± 3.6	32.4 ± 4.5	.005^+^*
Pre-op MT/L AVT			
Mean	1.7	1.3	.001*
Total operative time (min)			
Mean ± SD	304.7 ± 78.4	421.2 ± 101.7	.0034*^+^
Instrumented vertebrae			
Mean ± SD	7.7 ± 1.8	11.6 ± 3.9	.011*^+^
Hospital staying			
Mean ± SD	6.9 ± 2.5	8.1 ± 3.6	.088^+^
Post-op Coronal Cobb Angle (Mean ± SD)			
Main thoracic curve	14.3 ± 6.6	16.4 ± 9.9	.432
Lumbar curve	11.4 ± 5.4	13.9 ± 7.7	.521

*MT AVT* main thoracic apical vertical translation, *L AVT* lumbar apical vertical translation, *MT/L AVT* main thoracic/lumbar apical vertical translation

*Significant *p* value (Unpaired *t* test; *p* < 0.05)

^+^Unpaired *t* test

^§^Chi-square test

Table 2 shows the *p* values for the differences within groups at 24-month follow-up, compared with baseline. The SRS-22R mental health domain mean score showed a significant improvement in both Groups at 24-month follow-up, compared with baseline (Group A: *p* = 0.007; Group B: *p* = 0.003). In Group B patients, a significant improvement of SRS-22R self-image domain mean score was also depicted (*p* = 0.005), but a concomitant significant impairment of the SRS-22R function domain mean score was observed, at 24-month follow-up. Similar findings were depicted by the QLPSD score (Table 2). Therefore, Group B patients showed an improvement of the QLPSD body image domain mean score (*p* = 0.001) and a concomitant significant impairment of the QLPSD back flexibility domain mean score (*p* = 0.004), at 24-month follow-up. Moreover, a significant improvement of the SAQ mean scores was observed in Group B only, at 24-month follow-up.

**Table 2:** Patient-reported outcome measures for selective (Group A) and non-selective fusion (Group B) cohorts: *p* values for the differences within groups at 24-month follow-up, compared with baseline (Paired *t* test)

	Group A (selective fusion)			Group B (non-selective fusion)		
	Baseline Mean ± SD	24-month FU Mean ± SD	*p*	Baseline Mean ± SD	24-month FU Mean ± SD	*p*
SRS-22R Total	3.3 ± 0.5	3.6 ± 0.5	.114	3.3 ± 0.57	3.7 ± 0.5	.0965
SRS-22R Function	3.9 ± 0.4	3.7 ± 0.6	.553	3.8 ± 0.6	2.2 ± 0.5	.002*
SRS-22R Pain	3.7 ± 0.8	4.1 ± .0.4	.223	3.9 ± 0.9	4.3 ± 0.7	.446
SRS-22R Self-image	2.2 ± 0.6	2.5 ± 0.6	.721	2.3 ± 0.5	3.9 ± 0.4	.005*
SRS-22R Mental health	2.7 ± 0.7	4.1 ± 0.5	.007*	2.5 ± 0.4	4.2 ± 0.8	.003*
SRS-22R Satisfaction	NA	2.5 ± 0.8	-	NA	3.9 ± 0.4	-
QLPSD Total	48.1 ± 8.8	44.9 ± 9.9	.082	49.8 ± 8.7	46.1 ± 7.7	.082
QLPSD psychosocial functioning	1.9 ± 0.5	1.8 ± 1.1	.234	1.9 ± 0.6	1.8 ± 0.5	.545
QLPSD sleep disturbances	2.3 ± 0.7	2.2 ± 0.7	.345	2.4 ± 0.5	2.3 ± 0.9	.442
QLPSD back pain	2.8 ± 0.8	2.6 ± 0.8	.226	2.7 ± 0.8	2.6 ± 0.4	.653
QLPSD body image	2.4 ± 0.6	2.2 ± 0.9	.321	2.5 ± 0.5	1.3 ± 0.7	.001*
QLPSD back flexibility	2.1 ± 0.5	2.3 ± 0.5	.101	2.1 ± 0.4	3.1 ± 0.8	.004*
SAQ Total	50.3 ± 9.6	48.5 ± 8.8	.115	49.6 ± 10.6	30.3 ± 9.2	.001*
SAQ Appearance	32.9 ± 8.5	31.5 ± 7.5	.454	32.5 ± 7.2	20.2 ± 8.4	.001*
SAQ Expectations	18.2 ± 7.6	16.4 ± 4.4	.331	17.9 ± 9.7	9.8 ± 3.2	.001*

*SD* standard deviation, *FU* follow-up, *NA* not available

*Significant *p* value (*p* < 0.05)

**Fig. 1:**
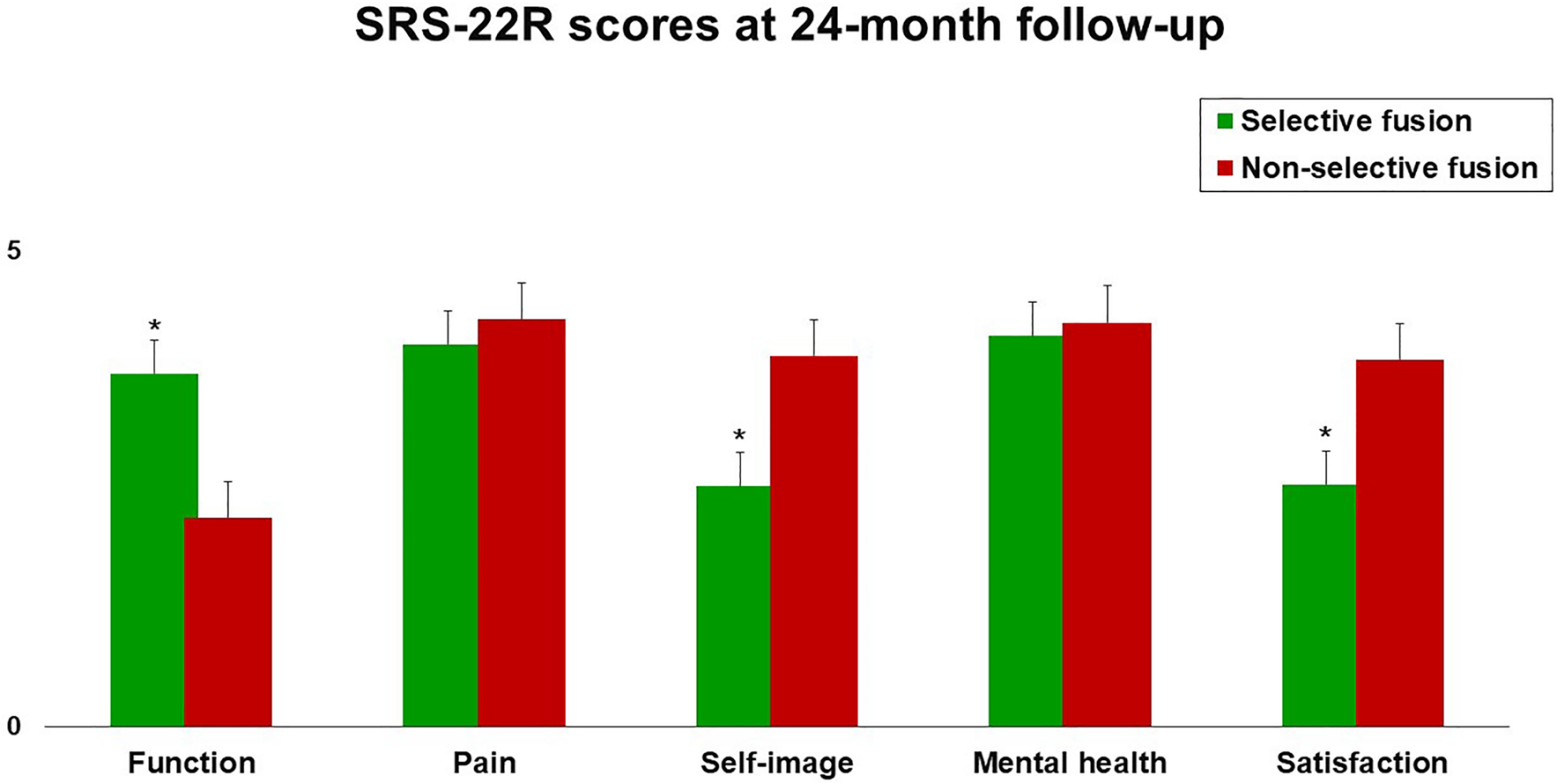
SRS-22 domains mean scores at 24-month follow-up in both groups. *p < 0.05 (unpaired *t* test)

**Fig. 2:**
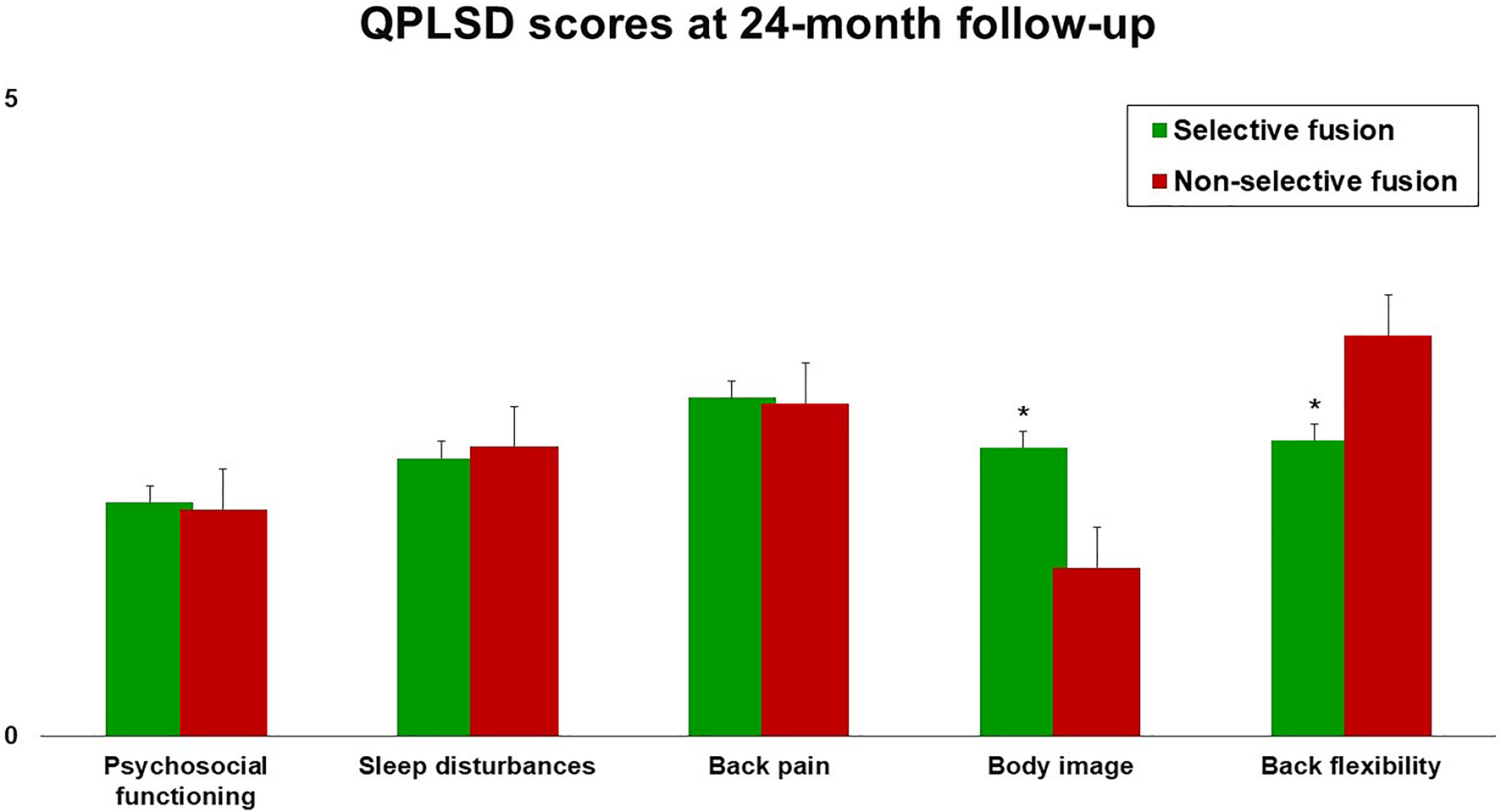
QLPSD domains mean scores at 24-month follow-up in both groups. *p < 0.05 (unpaired *t* test)

**Fig. 3:**
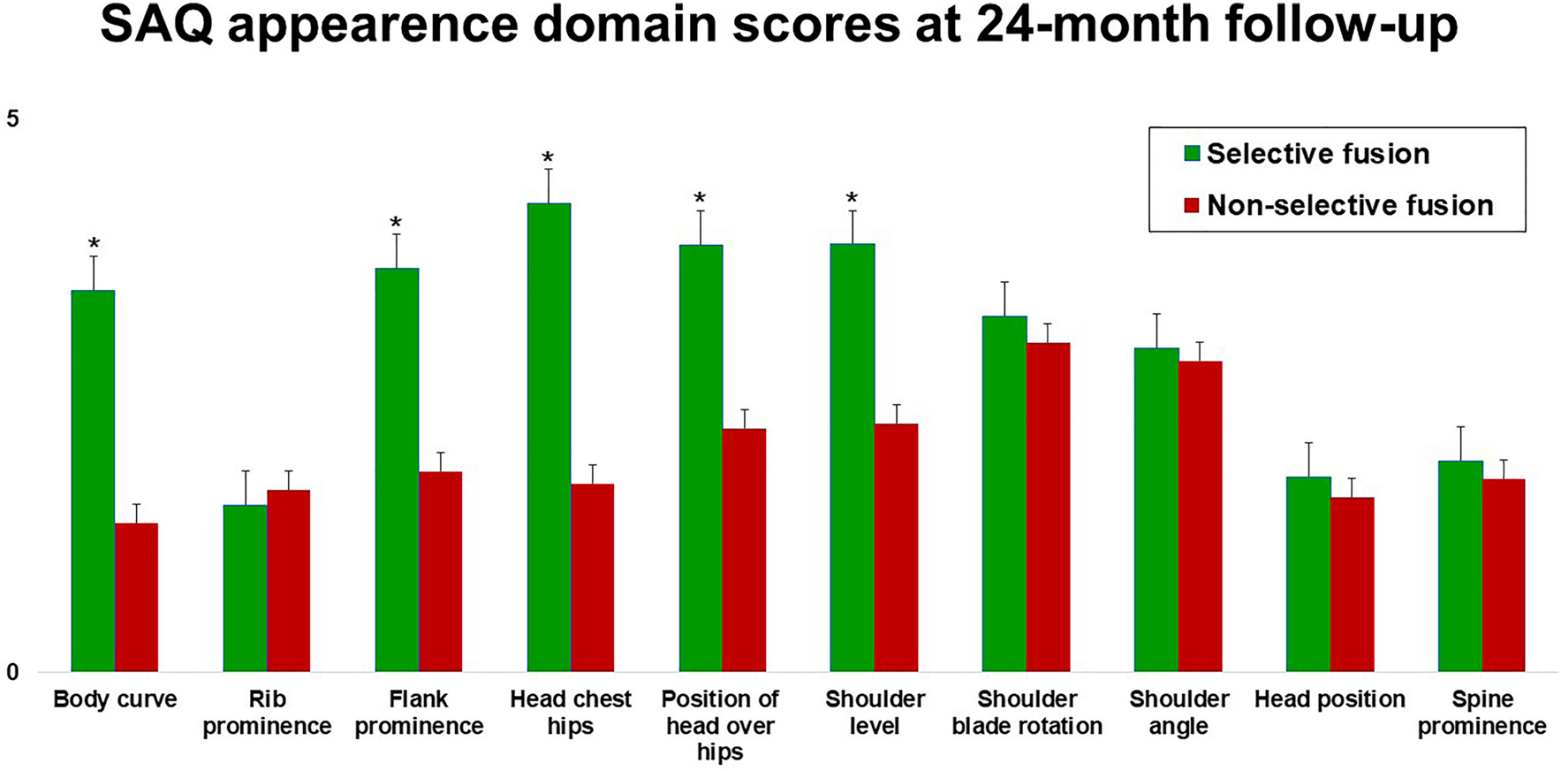
SAQ items mean scores at 24-month follow-up in both groups. *p < 0.05 (unpaired *t* test)

Table 3 shows the p values for the differences between groups at 24-month follow-up. Patients managed with N-STF (Group B) showed better SRS-22R self-image domain mean score (*p* = 0.012) and SRS-22R satisfaction mean score (*p* = 0.033), but worse SRS-22 function domain mean score (*p* = 0.006), compared with patients who underwent STF (Group A, Fig. 1). Similarly, Group B patients showed a significantly better QLPSD body image domain mean score (*p* = 0.005), but a worse QLPSD back flexibility domain mean score (*p* = 0.007), compared with Group A patients at 24-month follow-up (Fig. 2). Figure 3 shows SAQ appearance domain scores at 24-month follow-up in Group A compared with Group B. Group A patients showed significantly worse body curve (*p* = 0.003), flank prominence (*p* = 0.005), head chest hip (*p* = 0.001), the position of head over hips (*p* = 0.0001) and shoulder level (*p* = 0.002).

**Table 3:** Patient-reported outcome measures for selective (Group A) and non-selective fusion (Group B) cohorts: *p* values for the differences between groups at 24-month follow-up (Unpaired *t* test)

	Group A (Selective fusion) Mean ± SD	Group B (Non-selective fusion) Mean ± SD	*p*
SRS-22R total	3.6 ± 0.5	3.7 ± 0.5	.64
SRS-22R function	3.7 ± 0.7	2.2 ± 0.5	.006*
SRS-22R pain	4.1 ± .0.4	4.3 ± 0.7	.114
SRS-22R self-image	2.5 ± 0.6	3.9 ± 0.4	.012*
SRS-22R mental health	4.1 ± 0.5	4.2 ± 0.7	.784
SRS-22R satisfaction	2.5 ± 0.8	3.8 ± 0.4	.033*
QLPSD total	44.9 ± 9.9	46.1 ± 7.6	.221
QLPSD psychosocial functioning	1.8 ± 1.1	1.8 ± 0.5	.654
QLPSD sleep disturbances	2.2 ± 0.7	2.3 ± 0.9	.344
QLPSD back pain	2.6 ± 0.8	2.6 ± 0.4	.437
QLPSD body image	2.3 ± 0.9	1.3 ± 0.7	.005*
QLPSD back flexibility	2.3 ± 0.5	3.1 ± 0.8	.007*
SAQ total	48.5 ± 8.8	30.3 ± 9.2	.002*
SAQ appearance	31.5 ± 7.5	20.2 ± 8.3	.001*
SAQ expectations	16.4 ± 4.4	9.9 ± 3.2	.001*

*Significant *p* value (*p* < 0.05)

Table 4 shows the significant correlations (Pearson’s correlation test) between the number of instrumented levels and the Patient-Reported Outcome Measures at 24-month follow-up.

**Table 4:** Pearson’s Correlation Test between the number of instrumented levels and Patient-Reported Outcome Measures at 24-month follow-up (only significant correlations have been reported)

	24-months follow-up									
	SRS-22R self-image		QLPSD body image		SAQ Prominence		SAQ Trunk shift		SAQ Surgical scar	
	*R*	*p*	*R*	*p*	*R*	*p*	*R*	*p*	*R*	*p*
Number of instrumented levels	0.7	.004*	− 0.8	.001*	− 0.8	.001*	− 0.8	.001*	− 0.6	.03*

*Significant *p* value (*p* < 0.05)

Table 5 shows the Pearson’s correlation coefficient at 24-month follow-up between SAQ Appearance mean score, SRS-22R domains mean scores and QLPSD domain mean scores in Group A patients. A significant correlation was between SAQ appearance mean score and SRS-22R self-image (*R* = − 0.7), SRS-22R mental health (*R* = − 0.8), QLPSD psychosocial functioning (*R* = 0.7) and QLPSD back flexibility (*R* = 0.8) mean scores.

**Table 5:** Pearson’s Correlation Coefficient at 24-month follow-up between mean SAQ Appearance score, mean SRS-22R domains and mean QLPSD domain scores in patients undergoing selective thoracic fusion (Group A)

	SRS-22R Function	SRS-22R Pain	SRS-22R Self-image	SRS-22R Mental health	QLPSD psychosocial functioning	QLPSD sleep disturbances	QLPSD back pain	QLPSD body image	QLPSD back flexibility
SAQ appearence	-.213	-.316	− .721*	− .812*	.673*	.243	.227	.785*	.115

*Significant *p* value (*p* < 0.05)

## Discussion

Posterior STF for Lenke 1C AIS curves is a valid surgical option, aiming at achieving a good three-dimensional curve correction, while preserving spinal mobility and flexibility [8, 20]. Although STF has gained a growing interest in the last decades, currently, controversies still exist in its surgical indications and outcomes [7, 21].

Poor patient choice, wrong fusion-level selection and inadequate scoliosis curve correction have been related to relevant postoperative complications, including curve progression, shoulder imbalance, trunk shift and rotation worsening [1, 7, 8]. The correct patient choice mainly relies on the clinical examination features: patients with large lumbar rotational deformity and truncal shift to the left are poor candidates for STF [22]. Main thoracic curve (MTC) correction depends on the magnitude and the features of the lumbar curve, since, in selected cases, an under-correction might be needed, in selected cases, to promote the spontaneous postoperative lumbar curve correction [8, 12]. The selection of the fusion levels, on the other hand, is a challenging decision since it depends both on curve- and patients’ related factors—including the ratio between MTC and lumbar curve magnitude, trunk shift, skeletal maturity, body weight and activity level—but also surgeon’s-related factors, i.e., experience, surgical technique and achieved curve correction [12, 23, 24].

In recent years, significant progress has been made in the identification of radiographic parameters to aid surgeons in the decision between STF and N-STF. Bachmann et al., in a study analyzing 220 patients captured from a prospective longitudinal database, observed the lumbosacral takeoff angle, i.e., the angle between the center-sacral vertical line and a line through the centra of S1, L5, and L4 could be used to predict the residual lumbar Cobb angle in patients undergoing STFs [13]. Koller et al., in an interesting multicentre prospective database study recruiting 410 AIS patients, established an accurate prediction model for postoperative spontaneous lumbar curve correction, in patients undergoing selective STF [12]. Finally, Davies et al., in a retrospective comparative study recruiting 21 AIS patients, have found no significant radiological differences in lumbar curves between patients who underwent STF before and after menarche, at 2-year follow-up [14].

However, recent papers have paid little attention to the study of preoperative psychological, cosmetic and functional status, in patients undergoing STF or N-STF for AIS.

The present study has focused on the evaluation of the postoperative body’s self-image perception and QoL in 157 adolescent girls with Lenke 1C AIS curves, treated with posterior STF versus N-STF. At 24-month follow-up, SRS-22R mental health mean score showed a significant improvement in both groups (STF: *p* = 0.007; N-STF: *p* = 0.003), whereas SRS-22R self-image mean score (*p* = 0.005) and QLPSD body image mean score (*p* = 0.001) showed a significant improvement in patients treated with N-STF only (Table 2).

Patients managed with N-STF also showed significant worsening of SRS-22R function mean score (*p* = 0.553) and QLPSD back flexibility (*p* = 0.101). It is important to note, however, no significant changes in SRS-22R self-image mean score (*p* = 0.721) and QLPSD body image mean score (*p* = 0.234) were observed in female patients treated with N-STF. These data should be taken carefully into account in the decision-making between STF or N-STF for Lenke 1C AIS curves.

Comparisons between groups, at 24-month follow-up, showed significant worse SRS-22R self-image mean score (*p* = 0.012), QLPSD body image mean score (*p* = 0.005), but significant better SRS-22R function mean score (*p* = 0.006) and QLPSD back flexibility mean score (*p* = 0.007), in patients treated with STF, compared with N-STF.

SAQ appearance (*p* = 0.001) and expectation (*p* = 0.001) also revealed significantly worse mean scores in the STF group, compared with N-STF (Table 2). A detailed analysis of SAQ appearance items (Fig. 3) showed the recruited patients treated with STF had significant worse body curve (*p* = 0.003), flank prominence (*p* = 0.005), head chest hip (*p* = 0.001), the position of head over hips (*p* = 0.0001) and shoulder level (*p* = 0.002), compared to N-STF patients.

At 24-month follow-up, patients managed with STF showed a significant correlation between SAQ appearance mean score and SRS-22R self-image (*R* = − 0.7), SRS-22R mental health (*R* = − 0.8), QLPSD psychosocial functioning (*R* = 0.7) and QLPSD body image (*R* = 0.8).

The present findings confirm and better detail the data reported in previous studies [3, 8], thus highlighting the importance of a patient’s psychological evaluation before planning a posterior STF versus N-STF, in Lenke 1C AIS curves. Future studies will attempt to find correlations between preoperative psychological scores and unsatisfactory postoperative outcomes, in order the guide the decision-making process in patients with Lenke 1C AIS curves.

Furthermore, this study proposes some subjective identifiers in textual PROMs scales (SRS-22R and QLSPD) that significantly correlate with an unsatisfactory perceived postoperative body image, assessed with a pictorial scale (SAQ appearance), in adolescent female patients managed with STF, at 24-month follow-up.

Based on the reported findings, we highly encourage the routine use of SRS-22R, QLPSD and SAQ questionnaires in clinical practice, to correctly identify the patients that will have satisfactory results after STF.

One of the main limitations of this study is its retrospective nature. However, all the data were analyzed in a blind manner by an external statistician. Although we recruited a quite big number of patients, the lack of a power analysis is another limitation of the present study. The present study might be also affected by a potential selection bias, in the surgical strategy planning, since, as highlighted in the Results section, the two study groups showed a significantly different preoperative lumbar scoliosis curve Cobb’s angle.

Finally, future prospective studies with longer follow-up are needed to correlate the findings of the present study with long-term clinical and radiographic results.

## Conclusion

The selective thoracic fusion of Lenke 1C curves in adolescent patients provides better functional outcomes, but worse perceived body image, compared with N-STF, at 24-month follow-up.

The present study proposes some identifiers in textual PROMs scales (SRS-22R and QLSPD), that significantly correlate with unsatisfactory perceived body images, assessed with a pictorial scale (SAQ appearance), in adolescent patients managed with STF, at 24-month follow-up.

Based on the reported findings, particular attention should be addressed to preoperative patients’ mental health and body image perception, when choosing between STF and N-STF, in patients with Lenke 1C AIS.
